# Effectiveness of Atezolizumab in Addition to Chemotherapy in ES-SCLC: A Retrospective Real-World Monocentric Study

**DOI:** 10.3390/cancers17203298

**Published:** 2025-10-11

**Authors:** Raffaella Pagliaro, Fabiana Vitiello, Marina Gilli, Antonio d’Orologio, Luca Borgese, Susan F. Campbell, Paola Maria Medusa, Giuseppe Signoriello, Fabio Perrotta, Danilo Rocco, Andrea Bianco

**Affiliations:** 1 Department of Translational Medical Sciences, University of Campania Luigi, 80131 Naples, Italy; antonio.dorologio@studenti.unicampania.it (A.d.); luca.borgese@studenti.unicampania.it (L.B.); sfmcampbell@gmail.com (S.F.C.); paolamaria.medusa@unicampania.it (P.M.M.); fabio.perrotta@unicampania.it (F.P.); 2 Clinic of Respiratory Diseases “Vanvitelli”, Monaldi Hospital, A.O. dei Colli, 80131 Naples, Italy; 3 Department of Pneumology and Oncology, Monaldi Hospital, A.O. dei Colli, 80131 Naples, Italy; fabianavitiello@libero.it (F.V.); marinagilli@libero.it (M.G.); danilo.rocco@ospedalideicolli.it (D.R.); 4 Department of Mental and Physical Health and Preventive Medicine, University of Campania Luigi Vanvitelli, 80138 Naples, Italy; giuseppe.signoriello@unicampania.it

**Keywords:** atezolizumab, small cell lung cancer, real-world evaluation, immune check point inhibitors

## Abstract

*Background*: Small cell lung cancer (SCLC) is a malignant carcinoma characterized by high proliferative rate and early metastatization with limited treatment options and poor prognosis. The approval of ICIs has established a new standard of care for extensive-stage (ES)-SCLC (5). Atezolizumab, an anti PD-L1 monoclonal antibody, has been the first immune checkpoint inhibitor (ICI) to be approved for SCLC patients. This study aims to retrospectively evaluate the real-world effectiveness and safety of atezolizumab in a cohort of patients with ES-SCLC. *Methods*: We conducted a monocentric retrospective analysis of SCLC patients who received atezolizumab in addition to chemotherapy, between January 2020 and December 2023. Study design endpoints included progression-free survival (PFS), overall survival (OS), and adverse events. *Results*: A total of 134 patients were included in this study. Out of 134 patients who began the CEA protocol, 100 continued maintenance. Currently, 25 are alive, 17 still on atezolizumab, 5 on second-line therapy, and 3 receiving best supportive care. The median age was 65 years. Patients received a median of four cycles of CEA (range 1–6 cycles), while the median number of atezolizumab maintenance cycles was eight (range 0–75). The overall median survival was 15 months, with patients who received more than 30 cycles of atezolizumab showing OS of 46.7% at 48 months. Common adverse events included skin disorders, pneumonitis, colitis, alanine, and aspartate deaminase increment, dysthyroidism, and blood disorders with only 3% of patients experiencing grade 3 or higher toxicities. *Conclusions*: In this real-world cohort, atezolizumab demonstrated comparable effectiveness to clinical trial results, with a manageable safety profile. These findings support the use of atezolizumab as a viable treatment option for ES-SCLC in routine clinical practice.

## Simple Summary

Small cell lung cancer (SCLC) is an aggressive malignancy with limited treatment options and poor prognosis. Immune checkpoint inhibitors (ICIs), including atezolizumab, have been considered a new standard for extensive-stage (ES) SCLC. We retrospectively analyzed 134 ES-SCLC patients treated with atezolizumab plus chemotherapy from January 2020 to December 2023. Endpoints included progression-free survival (PFS), overall survival (OS), and safety profile. Median age was 65 years. Patients received a median of 4 chemotherapy cycles and 8 atezolizumab maintenance cycles. Median OS was 15 months; those receiving over 30 atezolizumab cycles had 46.7% OS at 48 months. Common adverse events included skin disorders, pneumonitis, and blood disorders, with only 3% experiencing grade ≥3 toxicity. Atezolizumab showed effectiveness and manageable safety consistent with clinical trials, supporting its use in routine ES-SCLC treatment.

## 1. Introduction

Small cell lung cancer (SCLC) is a high-grade neuroendocrine carcinoma which is strongly associated with cigarette smoking with a median age at diagnosis of 70 years old [1,2]. Accounting for about 15% of all lung cancer, SCLC remains one of the most aggressive cancers with a markedly high proliferative rate, poor prognosis mainly due to early metastatic spread, and cardio-respiratory complications leading to organ failure [3]. SCLC is traditionally classified into limited stage (LS) SCLC and extensive-stage (ES) SCLC. On a molecular setting, SCLC can be further divided into four distinct subtypes according to the differential expression of key neuroendocrine transcription factors [4,5]: subtype A (classic) characterized by high expression of ASCLC1, subtype N, marked by high expression of NEUROD1, subtype P, defined by increased POU2F3 expression, and subtype I (inflamed) which exhibits absent or low expression of neuroendocrine transcription factors. The ES defined as an extension of disease beyond a single radiation therapy field accounts for around 70% of patients with a median overall survival of roughly 10 months [6].

For decades, chemotherapy remained the mainstay of treatment for ES-SCLC with platinum plus etoposide representing the gold standard for treatment reaching a median overall survival (OS) of 9–10 months and a progression-free survival (PFS) of 5–6 months [7,8]. Since their introduction, immune checkpoint inhibitors (ICIs) have considerably improved patient life expectancy across several malignancies [7,8]; their combination with other therapeutic agents, including nanoparticles, has the potential to further enhance the efficacy [9].

More recently, the approval of ICIs has expanded therapeutic horizons for ES-SCLC establishing a new standard-of care [10,11]. Immune checkpoints play a physiological role in preventing T-cells from attacking healthy tissues by regulating inhibitory and stimulatory pathways [12,13].

Due to improved survival observed with ICIs, the standard of care first-line treatment of ES SCLC is the combination between anti PD-L1 and platinum–etoposide doublet chemotherapy [9,13]. Atezolizumab, a fully humanized monoclonal anti PD-L1 antibody, is the first immune checkpoint inhibitor (ICI) to be approved in combination with carboplatin and etoposide for the treatment of ES-SCLC [14,15,16].

The IMpower133 trial showed the first evidence of benefit from ICIs in ES-SCLC. The study demonstrated that the addition of atezolizumab, a PD-L1 inhibitor, to standard chemotherapy (carboplatin and etoposide) significantly improved both progression-free survival (PFS) and overall survival (OS) in patients who had not previously received treatment. Specifically, the median OS was 12.3 months with atezolizumab, compared to 10.3 months with chemotherapy alone (HR 0.70, *p* = 0.007), and the median PFS was 5.2 months vs. 4.3 months (HR 0.77, *p* = 0.02). The addition of atezolizumab also improved the 12-month OS and PFS rates, highlighting its efficacy in this patient population [17]. The IMbrella A extension study, which followed patients from IMpower133, reported a 5-year OS rate of 12% [18]. Recent study updates have indicated that the advantages of atezolizumab over placebo, along with its safety profile, were sustained at a median OS follow up of around 2 years [19,20].

Our real-world observational study aimed to evaluate clinical outcomes of ES-SCLC patients treated with Atezolizumab plus chemotherapy at the Department of Pneumology and Oncology at Monaldi Hospital in Naples. In particular, this study aimed to assess the efficacy of atezolizumab in addition to chemotherapy in terms of PFS and OS and whether the number of atezolizumab cycles administered affects survival outcomes. This study also aimed to provide an analysis of the safety profile of atezolizumab, including the frequency, types, and severity of adverse events, including immune-related adverse events (irAEs). This real-world study is essential for assessing whether the combination of atezolizumab and chemotherapy yields survival outcomes comparable to those reported in clinical trials.

## 2. Materials and Methods

### 2.1. Study Design

An observational retrospective study was conducted between January 2020 and December 2023. This study included 134 patients with diagnosis of ES-SCLC treated at the Department of Pneumology and Pneumology Oncology of the Monaldi Hospital in Naples. Patient demographics were collected, including clinical, pathologic, treatment, toxicity, and outcome data. The inclusion criteria were age >18 years, Eastern Cooperative Oncology Group (ECOG) performance status (PS) from 0 to 2, cytological or histological diagnosis of ES SCLC (stage IV), and patients eligible for therapy with atezolizumab. The exclusion criteria included the patients with a history of autoimmune disease, the absence of survival status, ECOG-PS > 2, patients with kidney failure, patients with insufficient clinical documentation, or unclear treatment history.

Clinical staging was determined based on Union for International Cancer Control Classification, Seventh Edition [20]. Patients received etoposide 80–100 mg/m^2^ (administered on days 1–3 of each 3-week cycle), the carboplatin area under the curve was 4 mg/mL/min (dosed on day 1 of each cycle), and atezolizumab 1200 mg was injected intravenously on day 1 of each 3-week cycle for 4–6 cycles in the induction phase, followed by atezolizumab maintenance every 3 weeks. Atezolizumab was used until one or more of the following conditions occurred as follows: unacceptable toxicity, disease progression, or death. According to the response evaluation criteria in solid tumors (RECIST) version 1.1 [21], tumor responses were classified as complete response (CR), partial response (PR), stable disease (SD), progressive disease (PD), and not evaluated (NE) every 4 cycles to evaluate the patient’s treatment effect and give corresponding treatment guidance. This study followed the common terminology criteria for adverse events (version 5.0) to assess drug treatment-related toxicity [22].

### 2.2. Data Source and Statical Analysis

Real-world data were gathered from consecutive patients treated for ES-SCLC at the oncology Department of the Monaldi Hospital in Naples, Italy. This study included patients with ES-SCLC who were eligible for atezolizumab based on the European Medicines Agency (EMA) monitoring registries. Baseline data collected included (a) demographic information, such as age (recorded at the time of the first atezolizumab administration) and sex, performance status (PS) based on the Eastern Cooperative Oncology Group (ECOG) PS scale, and the number and types of metastatic sites; (b) atezolizumab administration details, including dosage in milligrams, reasons and dates for discontinuing the drug, and the survival status of each patient. The baseline characteristics of patients were recorded. This study was conducted in accordance with the Declaration of Helsinki and the protocol was approved by the Local Department Ethics Committee. Informed consent was obtained from all subjects involved in the study. Moreover, PFS was defined as the time from the start of treatment to the occurrence of progressive disease (PD) or death from any cause and OS was measured from the initiation of treatment until death. Both PFS and OS were analyzed using the Kaplan–Meier method to perform survival curves according to number of cycles of atezolizumab and compared through the long-rank test. A *p*-value of less than 0.05 was considered statistically significant.

## 3. Results

### 3.1. Patient Characteristics

**Figure 1:**
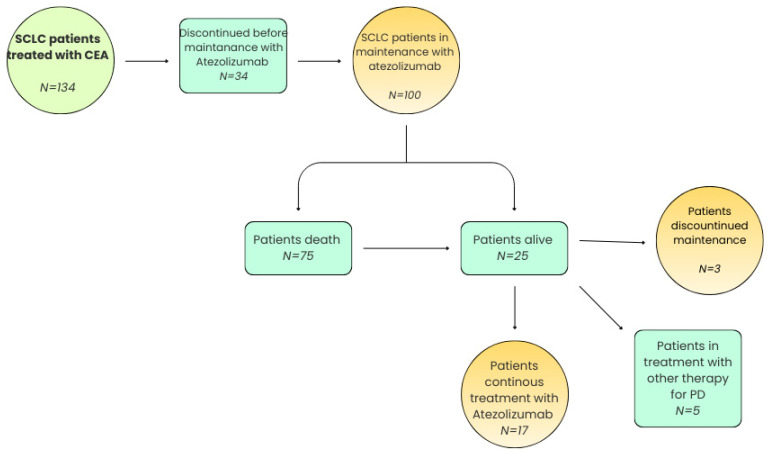
Patient selection diagram.

We enrolled 134 consecutive patients with diagnosis of SCLC admitted to the Department of Respiratory Disease, Pneumology, and Oncology at Monaldi Hospital between January 2020 and December 2023. These patients were treated by the initial combination of carboplatin, etoposide, and atezolizumab (CEA) for several cycles and atezolizumab was administrated as maintenance therapy. Of the 134 patients who initially started treatment with CEA protocol, 100 patients continued to the maintenance phase. Currently, 25 of 134 patients are alive, and among these, 17 patients are still undergoing treatment with atezolizumab as part of the maintenance regimen. A total of 5 patients have developed progressive disease (PD) and have been switched to second-line treatments, while 3 patients have transitioned to best supportive care (BSC), reflecting a shift in treatment goals. The enrolled patients are summarized in Figure 1.

The patients’ characteristics at baseline are described in Table 1. The study population consisted of 86 male (64.2%) and 48 female (35.8%). The median age of the cohort was 65 years (range 52–86). According to the ECOG-PS scale, 37 patients (27.6%) had a score of 0, 83 patients (61.9%) of 1, and 14 patients (10.4%) of 2. All patients had a smoking history, and, of these, 65 patients (48.5%) were current smokers. The median number of cycles for the combination of atezolizumab, carboplatin, and etoposide were 4 (ranging from 1 to 6) with two patients receiving 6 cycles and three patients receiving 5 cycles. In contrast, the median number of cycles of atezolizumab in maintenance therapy was 8 (range from 0 to 75) with 108 patients receiving fewer than 10 cycles.

We also divided our study population in four groups based on the number of atezolizumab cycles administrated as follows: <10 cycles, 10–20 cycles, 21–30 cycles, and >31 cycles. We then evaluated the correlation between these groups and several variables such as age, ECOG-PS, OS, PFS, and number of CEA cycles (Table 2). For each variable, the mean (± standard deviation) is provided, along with the *p*-value for the statistical comparison between the groups. The average age of patients in each group varies slightly but the *p*-value indicates no statistically significant difference in age between the groups. There is a significant difference in ECOG-PS between the group <10 cycles and 10–20 cycles; in particular, the <10 cycles group had more patients with worse performance status (ECOG 1 and 2). In contrast, the ECOG-PS 0 was more common in the >31 cycles group compared to the <10 patients’ group. The OS increases with the number of cycles of atezolizumab. Patients in the >31 cycles group had the longest survival (41.8 ± 2.1 months), while those in the <10 cycles group had the shortest survival (10.7 ± 8.8 months). The *p*-values for OS across groups are 0.001 between <10 cycles and 10–20 cycles, 0.116 between 21–30 cycles and >31 cycles, and <0.001 when comparing the <10 cycles group to the >31 cycles group, suggesting that a higher number of cycles is associated with improved OS. The PFS also increases with more cycles of atezolizumab. The >31 cycles group had the longest PFS (39.2 ± 1.6 months), while the <10 cycles group had the shortest PFS (6.9 ± 6.6 months). The *p*-values for PFS comparisons are <0.001 between the <10 cycles and >31 cycles groups, indicating that the number of cycles is significantly associated with PFS. For the average number of CEA cycles, the *p*-value <0.001 indicates a statistically significant difference between the groups in terms of the number of CEA cycles administered. The data suggests that more cycles of atezolizumab are associated with improved clinical outcomes, both in terms of OS and PFS. ECOG-PS and number of CEA cycles also play an important role in determining patient outcomes.

**Figure 2:**
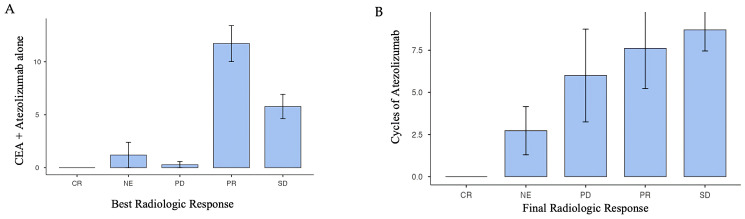
Treatment response. (**A**) evaluation of the best radiologic response to therapy (both CEA and atezolizumab therapy). (**B**) evaluation of the final radiologic response to atezolizumab therapy.

We also evaluated the correlation between number of cycles of atezolizumab and the radiologic response at CT scans, according to the RECIST criteria guidelines [21] divided into complete response (CR), partial response (PR), stable disease (SD), progressive disease (PD) and not evaluated (NE), as shown in Figure 2A,B below. A total of 11 patients were classified as not evaluated because they died before the first CT scan evaluation. During treatment, we observed that the most favorable radiologic response associated with the therapy was partial response (PR) while disease control, as indicated by stable disease (SD), was maintained with an average of 8.71 ± 11.5 cycles.

In our study, the toxicity profile of atezolizumab is illustrated in Table 3 and in Figure 2. In Table 3, immune-related adverse events (irAEs) were observed in a total of 31 patients with the most common adverse event being dysthyroidism and skin disorders. Notably, the most common adverse events were dysthyroidism (8 patients) and skin disorders (6 patients) while many of the adverse events were of moderate severity (grade 2) according to the Common Terminology Criteria for Adverse Events (CTCAE) version 5.0 [21]. There were a small number of severe cases of pneumonia and colitis with one patient experiencing severe pneumonia and two patients with severe colitis; liver enzyme elevations were also common. However, all patients were managed according to the established guidelines for the treatment of immunotherapy toxicity [22]; supportive therapies were administrated as needed.

**Figure 3:**
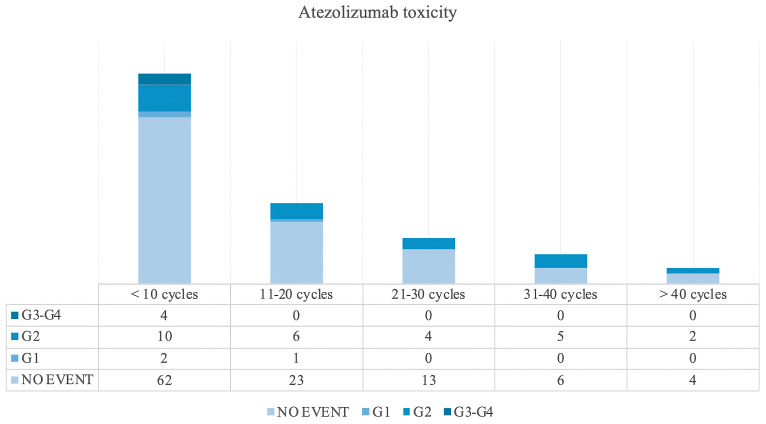
Most common treatment-related adverse events (TRAEs) reported in this study. The figure illustrates the relationship between the grade of toxicity and the number of cycles of atezolizumab administrated.

Overall, the adverse events seen in this study have been manageable and it was necessary to modify treatment only in a small percentage of cases. Figure 3 illustrates the relationship between the grade of toxicity and the number of cycles of atezolizumab administrated. According to our analysis, the frequency and severity of adverse events do not increase with a higher number of cycles. This suggests that, even with prolonged treatment, the risk of adverse effects remains relatively stable.

### 3.2. Survival Outcomes

**Figure 4:**
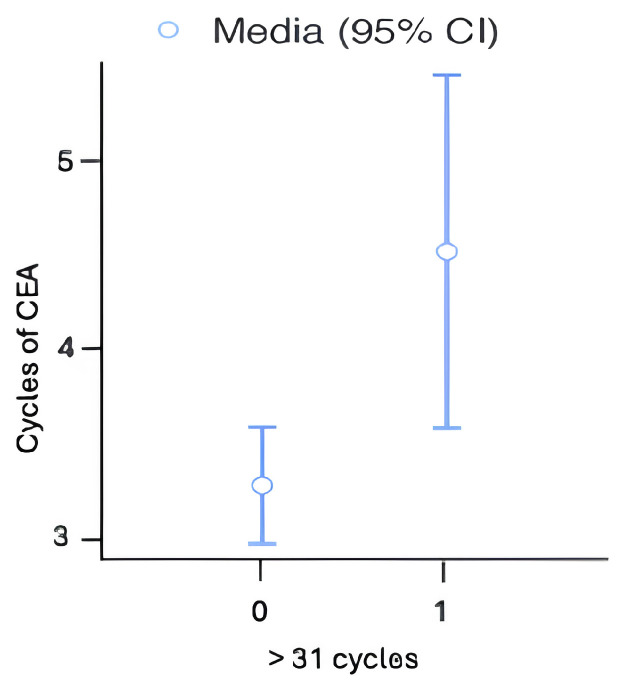
Correlation between cycles of CEA and >30 cycles of atezolizumab.

The median survival time was 15 months. A total of 10 patients received more than 31 cycles of atezolizumab and have a survival ≥30 months; in particular, nine of these patients have an OS of 36 months (Table 4). Moreover, we evaluated that patients who received more than 30 cycles of atezolizumab have a median of 4.30 ± 0.67 cycles of CEA as shown in Figure 4 below. Overall, 1-year, 2-year and 3-year cumulative survival rates were 51.5%, 21.7%, and 12.7%, respectively.

**Figure 5:**
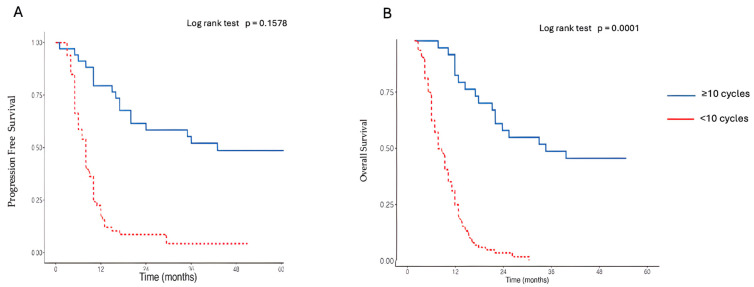
(**A**,**B**). Kaplan–Meier curves for progression-free survival (PFS) and overall survival (OS) by number of cycles of treatment received. In these figures, the blue lines represent patients who received fewer than 10 cycles of treatment while the red lines correspond to patients who underwent 10 or more cycles.

Furthermore, we evaluated the Kaplan–Meier survival analysis for PFS and OS based on the number of cycles of treatment received (Figure 5A,B below). The blue lines, indicating patients who received fewer than 10 cycles, show their PFS and OS rates. In contrast, the red lines, representing those who received 10 or more cycles, depict their corresponding survival outcomes.

**Figure 6:**
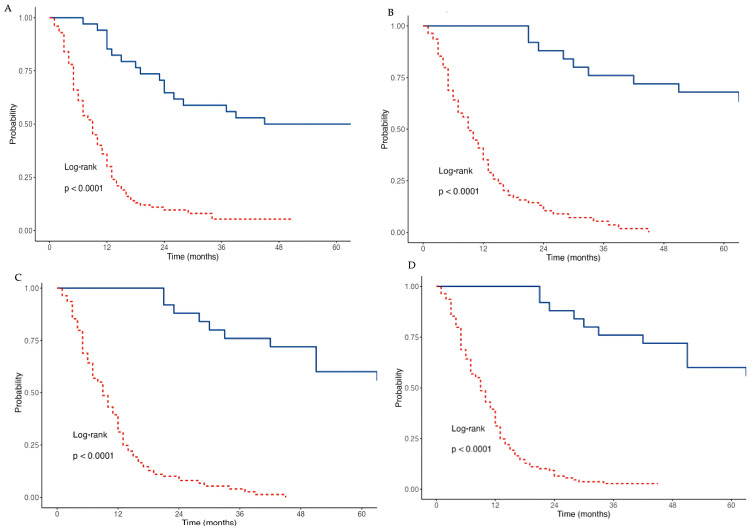
Kaplan–Meier curve for OS by number of cycles of atezolizumab administrated. The OS rates are shown for four groups based on the number of cycles of atezolizumab: <10 cycles (**A**), 10–20 cycles (**B**), 21–30 cycles (**C**), and >31 cycles (**D**). In each figure, the red lines represent patients who received the specified number of cycles according to the previous classification, while the blue lines indicate patients who received more than 10 cycles of atezolizumab.

We also assessed the differences in overall survival (OS) across the different groups based on the number of cycles of atezolizumab administered (Figure 6). In the group with <10 cycles, the OS was 30% at 12 months and 5.3% at 36 months. In the group receiving 10–20 cycles, the OS was 72.2% at 12 months and 38.9% at 36 months. In the group with 21–30 cycles, the OS was 57.1% at 36 months. Lastly, in the group with >31 cycles, the OS was 100% at 36 months, and at 48 months, it dropped to 46.7%.

According to univariate Cox regression, patients who received fewer than 10 cycles of atezolizumab showed a significantly higher risk of disease progression or death compared to the rest of the cohort. In this analysis, this group showed a hazard ratio of 5.45 (95% CI: 3.14–9.47; *p* < 0.001), indicating a markedly increased risk of the event.

Furthermore, we compared the presence of comorbidities at baseline and, in particular, the presence of respiratory failure and the most common sites of metastasis at the time of diagnosis (mediastinal, brain, liver, and bone) categorized by the number of treatment cycles received. A summary of these findings is shown in Table 5.

## 4. Discussion

In this retrospective study, we enrolled 134 patients with diagnosis of ES-SCLC treated with a combination of CEA followed by atezolizumab as maintenance therapy at a single center in Naples. The median age of our study population was 65 years (range 52–86). The median number of cycles for CEA was 4 with a range from 1 to 6 cycles. The median number of cycles for atezolizumab in the maintenance phase was 8 with a range from 0 to 75 cycles. In our population, the median survival rate was 15 months; patients who received more than 30 cycles of atezolizumab had the longest OS and PFS with 100% of OS at 36 months and 46.7% at 48 months. ECOG-PS and the number of CEA cycles used in the induction phase also influenced survival rates with worse outcomes observed in patients with higher ECOG-PS scores and fewer CEA cycles. Recent real-world data revealed that receiving six or more cycles of induction chemotherapy is a key indicator of improved prognosis. In particular, patients who received ≥6 cycles had significantly longer median PFS and OS compared to those who received fewer cycles (8 vs. 5 months and 18.5 vs. 13.1 months, respectively). Notably, subgroup analyses showed that the survival benefit was particularly evident in males, patients <65 years, and those with brain metastases [23].

The CASPIAN and IMpower133 trials, through post hoc analyses, determined that long-term survival was higher among patients receiving an ICI with standard chemotherapy as first-line therapy vs. chemo alone. In particular, in IMpower133, OS at 18 months was 34.0% for atezolizumab with CT (carboplatin) plus etoposide vs. 21.0% for CT and etoposide alone [24]. Interestingly, in an analysis post hoc of IMpower133, Liu et al. analyzed the association between different gene expressions and various subtypes of SCLC with long-term survival. In this study, a higher percentage of long-term survivors (LTS) were observed in the atezolizumab group (34%) compared to the placebo group (20%). The odds ratio for surviving 18 months or more was 2.1 in favor of the atezolizumab group (*p* < 0.03). Both groups of LTS showed enhanced immune-related signaling. Exploratory OS analyses indicated a treatment benefit of atezolizumab over placebo in subgroups defined by T-effector and B-cell gene signature expression. Additionally, a greater proportion of LTS compared to non-LTS in both groups had the SCLC-I subtype, with this difference being particularly notable in the atezolizumab group [19,25].

In the analysis from IMpower133, M. Reck et al. evaluated that maintenance with atezolizumab after induction with the atezolizumab plus chemotherapy improves benefits in terms of OS and PFS; these authors suggested that the administration of ICIs as induction therapy in combination with chemotherapy is relevant to the overall efficacy of ICIs as first-line therapy in ES- SCLC [19].

Regarding treatment tolerability, the safety profile of atezolizumab observed in our study is consistent with previously reported data. The most common adverse events included skin disorders, pneumonitis, colitis, alanine and aspartate deaminase increment, dysthyroidism, and blood disorders with 3% of patients experiencing grade 3 or higher toxicities. Notably, the incidence and severity of these adverse events were manageable and the frequency and severity did not significantly increase with the number of atezolizumab cycles; moreover, according to our population, nobody discontinued ICI treatment due to grade 5 of IRAs. This suggests that prolonged treatment does not exacerbate the risk of side effects. Close monitoring and early management of adverse events are central to allow the continuation of maintenance ICIs treatment. Patients who underwent increased numbers of atezolizumab demonstrated better OS.

In a recent analysis, the authors evaluated the toxicity profile of ICIs therapy across 14 clinical trials; they highlighted that patients with low-grade pneumonitis, cutaneous, and thyroid disfunction irAEs had better survival outcomes while patients with any grade of hepatitis did not show an improvement in terms of survival [26,27]. In another recent analysis, the authors evaluated the toxicity profile of ICIs therapy across 14 clinical trials; they highlighted that patients with low-grade pneumonitis, cutaneous, and thyroid dysfunction irAEs had better survival outcomes while patients with any grade of hepatitis did not show an improvement in terms of survival [26,27]. IrAEs are believed to result from T-cell activation with more severe irAEs, potentially reflecting increased T-cell activity which may be associated with better clinical outcomes [28]. However, previous studies on ICIs have not strongly demonstrated a clear relationship between irAEs severity and ICI efficacy, as severe side effects can lead to significant morbidity and mortality, complicating survival comparison [29]. To the best of our knowledge, this is the first study to include patients with SCLC who have received more than 30 cycles of atezolizumab (Table 5). These patients had baseline multi-organ comorbidities, including history of COPD, cardiovascular and metabolic disorders, and metastatic disease. Despite these factors, they showed favorable survival outcomes, likely due to the close monitoring they received. In particular, respiratory management plays a crucial role in optimizing their care, including regular respiratory monitoring and interventions, which are essential for preventing complications and maintaining stability [30]. Additionally, regular follow up visits are essential for managing potential toxicities, both related to immunotherapy and other causes.

Prompt management of adverse events increases the likelihood of preventing progression to grade 3/4 toxicities leading to irreversible discontinuing therapy. Another critical factor is organ failure, the management of which directly impacts performance status, potentially resulting in therapy interruption and a poorer prognosis. Toxicities and declining performance status not only limit treatment options but also have a detrimental effect on the patient’s overall life expectancy. Real-world studies are crucial for understanding the broader applicability of new therapies, as they include a more diverse patient population with varying comorbidities and performance statuses [31,32,33]. Our study’s population reflects a typical clinical practice scenario, providing a more comprehensive understanding of atezolizumab’s effectiveness and safety. These data can inform clinicians about the expected outcomes and potential challenges when prescribing atezolizumab in routine practice. According to a phase I/III trial, immune-related AEs were more frequent in the atezolizumab arm during both induction (28% vs. 17%; leading to atezolizumab/placebo interruption 9% vs. 5%, leading to withdrawal 4% vs. 0%) and maintenance (26% vs. 15%; leading to atezolizumab/placebo interruption, 3% vs. 2%, leading to withdrawal 1% vs. 1%), most commonly rash (induction 11% vs. 9%, maintenance 14% vs. 4%), and hypothyroidism (induction 4.0% vs. 0%, maintenance 10% vs. 1%) [34]. Lin Zhu et al. tried to find the predictive biomarkers of immunotherapy for ES-SCLC, such as PD-L1, tumor mutation burden (TMB), molecular subtypes, and gene expression; at present, the molecular subtypes defined from transcription factors may have some guiding significance, which still needs to be confirmed by prospective clinical studies [35]. Macrophages play a crucial role in the TME and a subtype (M2) was associated with anti-inflammatory and tumor-promoting properties. In advanced tumors, M2 tumor-associated macrophages (TAMs) predominate and serve as important regulators in response to the TME [36,37]. In this context, because of the immune heterogeneity of the cancer, four subsets of SCLC are identified in the population of patients of IMpower133. In particular, patients with low TAM and high T-effector signals had longer overall survival with PD-L1 blockade and CE vs. CE alone [38,39].

Our study has several limitations, including its retrospective design and the inherent biases associated with such analyses. The single-center nature of this study may limit the generalizability of the findings. Additionally, the relatively small sample size and lack of a control group restrict the ability to draw definitive conclusions. Prospective, multicenter studies with larger patient cohorts are needed to validate these findings and further elucidate the role of atezolizumab in SCLC treatment. Moreover, the MAURIS study provides preliminary evidence that the safety and efficacy of the IMpower133 regimen may be replicable in a population of ES-SCLC with more relaxed selection criteria, closer to that of “real life” clinical practice [20].

## 5. Conclusions

This real-world evaluation supports the use of atezolizumab as a beneficial treatment option for patients with ES-SCLC, demonstrating effectiveness and safety comparable to clinical trial results. Our findings in a real-world setting consolidate evidence supporting the role of immunotherapy in the treatment of ES-SCLC; this study emphasize the need for ongoing research to optimize patient selection, improving patient outcomes and survival. The effectiveness of current therapeutic approaches refines treatment strategies although better understanding of biomarkers is required to identify the potential long lasting responders. Future studies should focus on identifying predictive biomarkers for response and resistance to atezolizumab, as well as exploring innovative combination therapies to enhance its therapeutic efficacy.

## 6. Future Prospective

Recent studies suggest that the combination of IT with thoracic radiation (TRT) as a consolidation strategy could enhance therapeutic outcomes in patients with ES-SCLC [40]. In particular, radiation has been proposed to have immunomodulatory effects, including the potential of increasing tumor antigen production, enhancing antigen presentation and upregulate cytotoxic T-lymphocyte activity [41]. However, the safety and the benefits in terms of the impact of consolidative TRT in patients receiving CT + ICI regimens remain unclear [42]. In a retrospective analysis by Hoffmann et al., 41 patients with ES-SCLC were treated with TRT in 10 fractions of 3 Gy. The addition of TRT to the normal protocol significantly improved OS compared to systemic therapy alone (1-year OS 78.6% and 2-year OS 37.1% vs. 1-year OS 39.7% and 2 years not reached, *p* = 0.0019). Moreover, in patients receiving TRT upon progression, 1 year survival was 66.7%. In particular, the survival benefit was most pronounced when TRT was used as consolidation after initial CT-IT, compared to TRT used at the time of progression [43]. Furthermore, the Canadian consensus recommendations define practical guidelines regarding appropriate use of RT in the management of ES-SCLC; in this guidance, patients with a radiologic response to CT-IT, a good performance status, and limited metastases should be considered for consolidation treatment with TRT at a dose of 30 Gy; this treatment could be initiated during the phase of maintenance of IT [44]. The CREST trial conducted by Slotman et al. randomized patients with any response after 4–6 cycles of CT to receive TRT. Although the trial did not meet its primary endpoint of 1-year OS (33% in the TRT group vs. 28% in the control group), it reported a significant 2-year survival benefit with TRT (13% vs. 3%) [45]. A meta-analysis of the two randomized trials concluded that TRT improves both OS and PFS in patients with ES-SCLC [46]. Data from a multicenter study of the South of Italy provide additional support for using TRT as a consolidative strategy after CT-IT. In a cohort of 120 patients with ES-SCLC, none of them experienced disease progression after undergoing first-line CT-IT. Of these, 59 patients received consolidative TRT. Notably, only 10% of patients receiving TRT experienced radiation toxicity with no grade ≥ 3 radiation-induced adverse events. The median time for the completion of CT-IT to TRT was 62 days. When compared to systemic therapy alone, consolidative TRT was associated with a significantly longer PFS (1-year PFS of 61% vs. 31%, *p* < 0.001) and a trend toward improved OS (1-year OS of 80% vs. 61%, *p* = 0.027) [47]. In conclusion, consolidative TRT shows promise as a strategy to improve survival in patients with ES-SCLC. Further prospective studies are essential to establish its role in combination with IT and its impact on treatment strategies.

Recent advances in the treatment of ES-SCLC include the approval of lurbinectedin for the second-line therapy [48]. Lurbinectedin induces cancer cell apoptosis by binding to the minor groove of DNA and inhibiting RNA polymerase II, thereby blocking gene transcription [49]. IMforte, a phase III, randomized, open-label, multicenter study is investigating its use in combination with atezolizumab as maintenance therapy. This trial compares the combination to atezolizumab alone in patients who have completed first-line induction therapy with carboplatin, etoposide, and atezolizumab. Eligible patients must be treatment-naïve for ES-SCLC or have been treatment-free for at least six months following prior curative-intent therapy for limited-stage disease in patients with ES-SCLC. Eligible participants must have received no prior systemic therapy for ES-SCLC or have been treatment-free for at least six months following curative-intent therapy for limited-stage disease. This study is ongoing and currently in the enrollment phase.

Additional clinical trials have identified DLL3 inhibitors as a promising therapeutic approach in SCLC [50]. The anti-DLL3 antibody, tarlatamab, targeting bispecific T-cell engagers, has shown encouraging results in terms of objective response and safety, and also in the presence of brain metastases [51]. Several PARP inhibitors have been investigated in SCLC clinical trials [52] without definitive results in improving survival outcomes [53]. However, combination strategies with other agents have achieved satisfying results in terms of therapeutic effectiveness based on specific patient selection and biomarker-driven approaches [29]. Also, CHK1 inhibitors, especially prexasertib, have been investigated, exhibiting limited efficacy as monotherapy in ES-SCLC [54], whilst combining CHK1 inhibitors with other agents like lurbinectedin or PARP inhibitors may enhance antitumor activity [55].

## Figures and Tables

**Table 1:** Baseline patient characteristics enrolled in our retrospective study.

Characteristic	Tot. Patient
Patients, n.	134
Age, yr, median (range)	65 (52–86)
Male	86 (64.2%)
Female	48 (35.8%)
ECOG-PS	
0	37 (27.6%)
1	83 (61.9%)
2	14 (10.4%)
Smoking status	
Yes	134 (100%)
Ex	65 (48.5%)
Brain metastasis at diagnosis	18 (13.5%)
Mediastinal metastasis at diagnosis	89 (66.4%)
Liver metastasis at diagnosis	37 (27.6%)
Bones metastasis at diagnosis	39 (29.1%)
N. cycles of CEA	
1	8 (6%)
2	6 (4.5%)
3	12 (9%)
4	103 (76.9%)
5	3 (2.2%)
6	2 (1.5%)

**Table 2:** A comprehensive comparison of different variables across different groups based on the number of atezolizumab cycles administrated. This analysis includes the evaluation of age, performance status, OS, PFS, and number of CEA.

	<10 Cycles	*p*-Value	10–20 Cycles	*p*-Value	21–30 Cycles	*p*-Value	>31 Cycles	*p*-Value
Age	65.2 ± 7.4	0.225	67.2 ± 11	0.357	61 ± 6	0.753	67.8 ± 5.7	0.741
ECOG-PS		<0.001		<0.001		0.741		0.056
0	24		8		2		3	
1	64		10		5		4	
2	12		1		0		1	
OS	10.7 ± 8.8	<0.001	20.7 ± 9.2	0.001	28.7 ±2.55	0.116	41.8 ± 2.1	0.016
PFS	6.9 ± 6.6	<0.001	15.8 ± 6.7	0.004	20.2 ± 2.23	0.089	39.2 ± 1.6	0.038
Number of CEA	3.6 ± 0.9	<0.001	3.9 ± 0.8	<0.001	4.2 ± 0.9	<0.001	4.4 ± 0.9	<0.001

OS: overall survival; PFS: progression-free survival. CEA: carboplatin, etoposide, atezolizumab.

**Table 3:** Immune-related adverse events (irAEs) in patients undergoing maintenance therapy with atezolizumab.

	Any Grade	Grade 5	Grade 3 or 4	Grade 2	Grade 1
Any event	33	0	4	26	3
Pneumonia	5	0	1	4	0
Dysthyroidism	8	0	0	7	1
Skin disorders	6	0	0	6	0
Colitis	4	0	2	2	0
AST and ALT increased	6	0	0	4	2
Edema legs	2	0	0	2	0
Bloods and lymphatic disorders	2	0	1	1	0

**Table 4:** Evaluation of patients according to number of cycles of treatment with atezolizumab as maintenance.

Number of Cycles of Atezolizumab as Maintenance	Number of Patients
<10 cycles	108
11–20 cycles	11
21–30 cycles	7
31–40 cycles	3
>41 cycles	5

**Table 5:** Distribution of baseline comorbidities, respiratory failure, and common metastatic sites at diagnosis according to the number of treatment cycles received.

	<10 Cycles	10–20 Cycles	21–30 Cycles	>31 Cycles	Tot.
Comorbidities at baseline	72 (53.8%)	4 (3.0%)	3 (2.3%)	8 (6.0%)	87 (65.1%)
Respiratory failure at baseline	16 (11.9%)	0 (0%)	3 (2.2%)	3 (2.2%)	22 (16.3%)
Metastasis at baseline:					
Mediastinal	84 (62.8)	16 (12%)	6 (4.5%)	8 (6.0%)	114 (85.3%)
Cranial	9 (6.7%)	1 (0.8%)	4 (3.0%)	2 (1.5%)	16 (12.0%)
Liver	22 (16.4%)	7 (5.2%)	1 (0.8%)	0	30 (22.4%)
Bone	27 (20.2%)	7 (5.2%)	3 (2.5%)	4 (3.0%)	41 (30.9%)

## Data Availability

No new data were created or analyzed in this study. Data sharing is not applicable to this article.
